# Cyanopeptide Mixtures Induce Variable Synergistic and Antagonistic Effects Across Diverse Human Cell Lines

**DOI:** 10.1002/tox.70028

**Published:** 2026-01-10

**Authors:** Lauren N. Hart, Katherine L. Lev, Sierra Hefferan, Sanduni H. Premathilaka, Sharmila I. Thenuwara, Dragan Isailovic, David H. Sherman, Gregory J. Dick

**Affiliations:** ^1^ Program in Chemical Biology University of Michigan Ann Arbor Michigan USA; ^2^ Life Science Institute University of Michigan Ann Arbor Michigan USA; ^3^ Department of Medicinal Chemistry University of Michigan Ann Arbor Michigan USA; ^4^ Department of Chemistry and Biochemistry University of Toledo Toledo Ohio USA; ^5^ Department of Chemistry University of Michigan Ann Arbor Michigan USA; ^6^ Department of Microbiology & Immunology University of Michigan Ann Arbor Michigan USA; ^7^ Department of Earth and Environmental Sciences University of Michigan Ann Arbor Michigan USA; ^8^ Cooperative Institute for Great Lakes Research, School for Environment and Sustainability University of Michigan Ann Arbor Michigan USA

**Keywords:** A549 cells, anabaenopeptin, cyanopeptides, cyanotoxins, cytotoxicity, Hep‐3B cells, HK2 cells, microcystin, mixture effects

## Abstract

Cyanobacterial harmful algal blooms (cyanoHABs) threaten human, animal, and ecosystem health and safety through production of toxic secondary metabolites. *Microcystis,* a cosmopolitan bloom‐forming cyanobacterial genus, is well‐known for producing hepatotoxic microcystins (MCs), but it can produce many other bioactive cyanopeptides, such as anabaenopeptins (APs), that occur at high levels in blooms. The toxicological and ecological impacts of such co‐occurring cyanopeptides in the natural environment remain understudied. Here we evaluated the effects of pure MCs and APs individually and in combination, as well as extracts of *Microcystis* cultures producing diverse suites of cyanopeptides, including strains with and without MCs and APs, on human lung (A549), kidney (HK2), and liver (Hep‐3B) cell viability. Individual MC and AP congeners exhibited a gradient of toxic effects across cell lines; MC‐LA caused the most toxic effects, MC‐LR had comparable effects to AP‐A and AP‐B, and MC‐RR caused the least toxicity. Combined exposure to MC‐LA and AP‐B produced dose‐dependent synergistic effects across all three cell lines. *Microcystis* culture extracts significantly reduced cell viability in dose‐ and *Microcystis* strain‐dependent patterns that could not be explained by microcystin or anabaenopeptin content alone, suggesting a role of other metabolites and their interactions within the mixtures. These findings demonstrate that mixtures of environmentally relevant cyanopeptides can have greater toxic threats than individual compounds and underscore the importance of considering metabolites beyond MCs and their potential interactions in public health, future risk assessments, and management strategies for cyanoHAB‐impacted waters.

## 1 Introduction

Cyanobacterial harmful algal blooms (cyanoHABs) are a growing, global threat to freshwater ecosystems and public health due to their production of toxic secondary metabolites [1, 2, 3, 4]. Among these, only four cyanotoxins are currently monitored by federal agencies in the United States: microcystins (MCs), cylindrospermopsin, anatoxin‐a, and saxitoxin [5, 6, 7]. These compounds can accumulate to micromolar concentrations in freshwater systems used for drinking, recreation, and agriculture, posing acute and chronic health risks to humans and animals depending on their structure, exposure route and duration, mode of entry, and mechanism of action [8, 9, 10, 11]. However, these four cyanotoxins and their related congeners represent only a small fraction, approximately 10%, of the more than 3000 cyanobacterial metabolites identified to date [12, 13]. Many of these unmonitored cyanobacterial metabolites exhibit diverse and potent bioactivities, yet remain poorly characterized, particularly in the context of human health risk [14, 15]. Beyond the known and predicted risks of characterized cyanobacterial metabolites, advances in metabologenomics suggest that a wealth of uncharacterized cyanobacterial secondary metabolites remain undiscovered, representing an even greater, largely unassessed toxicological unknown [16, 17].

**FIGURE 1:**
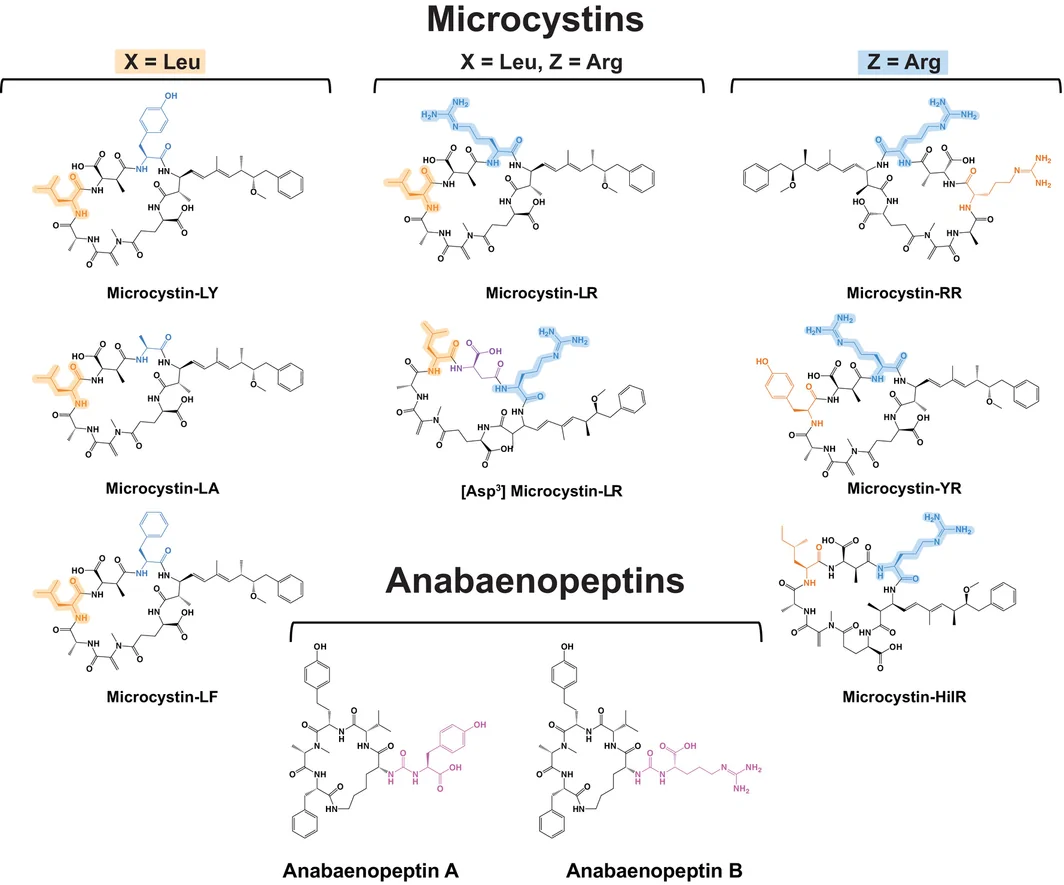
Chemical structures of microcystins (LY, LA, LF, LR, Asp^3^‐LR, RR, YR, and HilR) and anabaenopeptins A and B. Microcystins are grouped based on amino acid substitutions at the variable *X* and *Z* positions: Leucine (Leu) residues at position *X* are highlighted in orange, and arginine (Arg) residues at position *Z* are highlighted in blue. Structural differences between anabaenopeptins A and B are shown in magenta.

MCs are the most well‐studied class of cyanobacterial metabolites and are synthesized via a hybrid nonribosomal peptide synthetase‐polyketide synthase (NRPS‐PKS) biosynthetic pathway [18]. They are cyclic heptapeptides with over 300 characterized congeners produced by various bloom‐forming cyanobacterial genera, including *Microcystis*, *Dolichospermum*, *Planktothrix*, *Anabaena*, and *Oscillatoria* [12, 13, 19, 20]. Their structure consists of seven amino acids, including the distinctive Adda moiety [(2S,3S,8S,9S)‐3‐amino‐9‐methoxy‐2,6,8‐trimethyl‐10‐phenyldeca‐4,6‐dienoic acid], which is critical for hepatotoxicity [18, 21]. MC congener diversity predominantly arises from variations at the second (*X*) and fourth (*Z*) amino acid positions (Figure 1). Despite structural diversity, all MCs potently inhibit serine/threonine protein phosphatases PP1 and PP2A in the low‐nanomolar range in vitro, leading to disruption of cytoskeletal integrity and cell signaling pathways [22, 23, 24, 25]. Their toxic potential in vivo, however, varies widely by congener (e.g., 50–600 μg/kg in mice) [26, 27]. The discrepancy between in vitro PP inhibition and in vivo toxicity may be explained by differences in cellular uptake for distinct congeners via organic anion transporting polypeptides (OATPs), primarily OATP1B1 and OATP1B3 [28, 29, 30, 31]. These transporters mediate the active entry of amphiphilic MCs into hepatocytes and other tissues, and OATP inhibitors ameliorate the hepatoxic effects of MCs [32, 33, 34, 35]. In vitro toxicology studies of MCs must therefore account for cell line‐specific OATP expression. In addition to PP inhibition, MC exposure has been linked to damage in liver, kidney, lung, and skin tissues, induction of inflammatory responses, and oxidative stress [36, 37, 38].

*Microcystis* is one of the most common and abundant bloom‐forming cyanobacteria globally and is studied extensively for its ability to produce MCs [1, 39]. However, diverse *Microcystis* strains also produce strain‐specific mixtures of other bioactive cyanopeptides, including anabaenopeptins (APs), cyanopeptolins, microviridins, microginins, aeruginosins, and cyclamides [14, 16, 40]. These metabolites target diverse enzymes, such as serine proteases, peptidases, and phosphatases, and likely influence bloom ecology through allelopathy and grazing deterrence, yet only ~10% of studies on *Microcystis* secondary metabolism focus on these compounds [15]. Recent monitoring efforts suggest that several non‐MC cyanopeptides, including APs, are often present at concentrations exceeding those of MCs during bloom events, raising concerns about their individual and combinatorial roles in human exposure and toxicity [8, 41, 42, 43, 44, 45].

APs are cyclic hexapeptides synthesized via a NRPS biosynthetic pathway and are structurally characterized by an invariant lysine and a ureido linkage with over 120 AP congeners characterized to date [12, 13]. AP concentrations in environmental samples can reach levels greater than 1000 μg L^−1^ in freshwater systems, with AP‐A, AP‐B, and AP‐F among the most commonly detected congeners [8, 46]. While APs inhibit PP1 and PP2A in the mid to low micromolar range, they potently inhibit carboxypeptidases at low‐nanomolar concentrations [47, 48]. Despite their demonstrated bioactivity, APs remain unregulated, are not routinely monitored, and are still broadly categorized as nontoxic. One of the few in vivo studies on APs to date demonstrated that AP‐A, B, and F reduced growth, hatching, reproduction, and lifespan in nematodes ( *Caenorhabditis elegans* ) to a similar extent as MC‐RR [49]. However, their mode of entry, mechanism of action, subcellular localization, and clearance in human models remain uncharacterized.

While MCs and APs are frequently detected together in environmental samples, their combined toxicity has not been extensively studied in human‐relevant systems. Evidence from *Daphnia* and trout suggests that MC–AP combinations exert synergistic toxic effects, raising concerns that co‐exposure in humans and wildlife may also result in enhanced toxicity [50, 51]. Fish, such as bass, trout, and pike, have been shown to accumulate both MCs and APs, implying potential dietary exposure risks [52]. Further, the presence and concentration of MCs in *Microcystis* extracts often poorly predict toxicity, suggesting that additional, unidentified metabolites or interactions among metabolites are driving biological effects [53]. These combinatorial effects may stem from multiple mechanisms, including additive or synergistic biochemical activity, competitive or facilitated uptake, or modulation of metabolic and signaling pathways [54]. Yet, few studies have systematically explored these dynamics in human cell models.

In this study, we investigated the cytotoxic effects of environmentally relevant MC and AP congeners individually, in combination, and within complex *Microcystis* metabolite mixtures on human lung (A549), kidney (HK2), and liver (Hep‐3B) cell lines. By comparing dose‐ and congener‐dependent effects across cell types and assessing the effects of *Microcystis* metabolite mixtures, we aimed to uncover how combinatorial exposure shapes toxicity in human‐relevant systems.

## 2 Materials and Methods

### 2.1 Cell Line Rationale

In this study, we selected to use A549 (lung), HK2 (kidney), and Hep‐3B (liver) human cell lines based on the known impacts of cyanotoxins and bioactive cyanopeptides on these organs. Each cell line expresses at least one OATP transporter [38, 55, 56], making them appropriate models for evaluating the toxicity of microcystins (MCs) [29, 57].

### 2.2 Cell Lines and Maintenance

Human hepatocellular carcinoma (liver epithelial) Hep‐3B cells, human adenocarcinoma (lung tissue) A549 cells, and human immortalized kidney HK‐2 cells were acquired from ATCC (Virginia, USA, catalog no. HB‐8064) [58]. Cells were cultured under standard conditions according to ATCC recommendation and stored in Gibco Recovery Cell Culture Freezing Medium at 2 × 10^6^ cells per aliquot. Cells were cultured in Dulbecco's Modified Eagle Medium (Hep3B cells and HK2 cells, DMEM, Life Technologies media, Carlsbad, CA, USA) or F‐12K medium (ATCC, Virginia, USA) supplemented with 10% fetal bovine serum (FBS) and 1% penicillin/streptomycin. Cultures were maintained at 37°C with 5% CO_2_. All cell lines were tested to ensure they were mycoplasma‐free and were separately verified through short tandem repeat profiling, conducted by ATCC. No cell lines were previously reported as misidentified or contaminated through NCBI databases.

### 2.3 *Microcystis* Culturing

Seven *Microcystis* cultures from the western Lake Erie culture collection (WLECC) were selected for analysis in this study. Cultures were selected based on diversity of phylogeny [59] and biosynthetic potential [16] and their ability to grow in 500 mL volumes. The cultures selected were LE18‐22.4, LE19‐55.1, LE19‐131.1, LE19‐41.2, LE17‐20, LE19‐197.1, and LE19‐84.1. Cultures were grown without shaking at 25°C at 50 μmol photons m^−2^ s^−1^ light intensity on a 12:12 h diel cycle in BG11‐2N media [58]. All cultures were started in a 10 mL volume and each culture was grown in triplicate to 500 mL. One replicate from the strains LE19‐41.2 and LE19‐84.1 bleached during growth and resulted in these cultures only being grown in duplicate for the study. Uninoculated media controls were maintained in triplicate within the same incubator.

### 2.4 *Microcystis* Culture Biomass Collection and Metabolite Extraction

Biomass was harvested after all strains were maintained at 500 mL for 1 week. Cultures were centrifuged, supernatant was decanted, and cell pellets were collected using a sterile metal scoopula. Cell pellets were put into glass scintillation vials and frozen at −20°C until extraction. Metabolites were extracted from the cellular biomass using a freshly made 50:50 high‐performance liquid chromatography (HPLC)‐grade methanol/chloroform mixture. For each sample, two clean, pre‐weighed vials, one for cell viability assays and one for targeted metabolomics analysis, were prepared. Extraction mixtures were filtered to remove residual solids and then evenly divided between the two vials. After filtration, vials were dried under N_2_ gas. Dried samples were re‐weighed to calculate their crude extract weight and stored at −20°C until further analysis.

### 2.5 Targeted Metabolomics Data Collection and Analysis

Targeted metabolomics vials for each sample were delivered to the University of Toledo on ice and were stored in −20°C prior to analysis. Microcystin and anabaenopeptin congener composition was determined using liquid chromatography‐mass spectrometry (LC–MS) via methods previously described in Baliu‐Rodriquez et al. [20] and Flores and Caixach [41], respectively. Microcystin and anabaenopeptin congener identification was confirmed via standards for MC‐LR, MC‐RR, MC‐YR, MC‐LA, MC‐LF, [Asp^3^] MC‐LR, MC‐HilR, MC‐LY, AP‐A, and AP‐B (Figure 1). Characteristic fragment ions used to identify MC congeners by tandem mass spectrometry (MS/MS) were *m*/*z* 135.08 [C_6_H_5_—CH=CH—OCH_3_ + H]^+^ (from Adda) and 213.09 [Glu‐Mdha + H]^+^, and the diagnostic fragment ion for APs was 84.08 *m*/*z* [Lys + H‐CO‐NH_3_]^+^. If an AP contains a C‐terminal Arg, it shows additional characteristic fragment ions 201.09 *m*/*z* [CO + Arg—COOH]^+^ and 175.11 *m*/*z* [Arg—COOH + 2H]^+^.

### 2.6 Cell Line Exposures to Individual Cyanopeptide Standards

**FIGURE 2:**
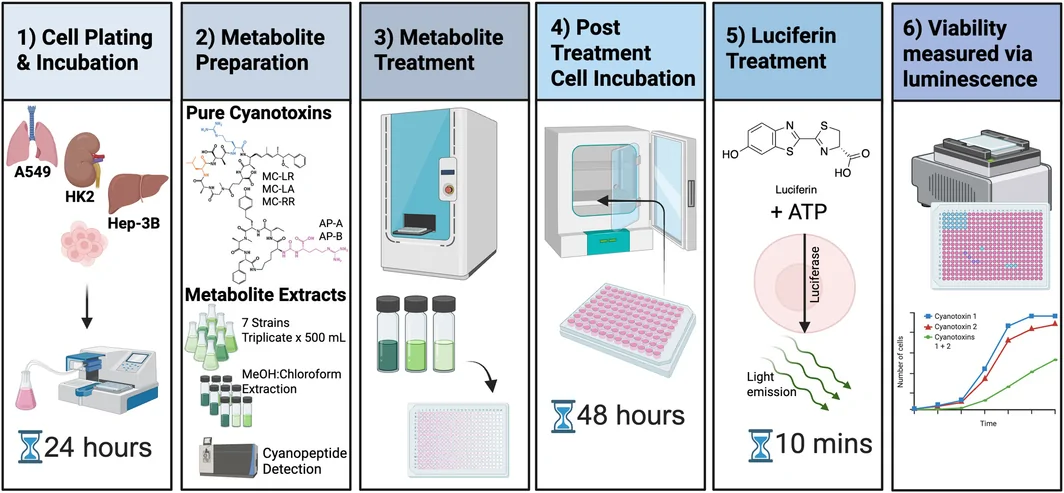
Workflow for preparing cyanopeptide standards and culture‐derived metabolite extracts and assessing their effects on cell viability in human cell lines using the CellTiter‐Glo assay. Pure standards (MC‐LR, MC‐LA, MC‐RR, AP‐A, AP‐B) and extracts from seven WLECC *Microcystis* isolates were prepared in biological triplicate. After metabolite extraction and cyanopeptide quantification, human cell lines (A549, HK‐2, and Hep‐3B) were plated and incubated for 24 h, followed by toxin treatment and a 48‐h incubation. Cell viability was measured via luminescence.

Pure cyanopeptide standards in powder form were purchased from Cayman Chemical (Ann Arbor, MI, USA) (MC‐LR) and Enzo Life Sciences (Farmingdale, NY, USA) (MC‐LA, MC‐RR, AP‐A, AP‐B). Doxorubicin was purchased from AK Scientific (Union City, CA, USA) in powder form and used as a positive control in all assays. Powder stocks were stored at 4°C, and liquid stocks were made in dimethyl sulfoxide (DMSO) and stored at −20°C for the duration of experimental use. The viability and toxicity of cyanopeptide standards individually were assessed in Hep‐3B, A549, and HK‐2 cells using the CellTiter‐Glo Luminescent Cell Viability Assay according to manufacturer's instructions (Promega, Madison, WI, USA). Cells were seeded in 100 μL of medium at a density of 10 000 cells per well in opaque, 96‐well tissue culture‐treated plates after reaching desired confluency and viability > 90% in flasks and allowed to adhere for 24 h. The A549 cell line was seeded at 4000 cells per well due to the faster doubling time. After adherence, the culture media was replaced with fresh medium, and cells were treated with two‐fold serial dilutions of single cyanopeptides and doxorubicin, with the highest concentration being 100 μM. The plates were incubated at 37°C for 48 h. After incubation, cell viability was determined by luminescence reading (Agilent BioTek Microplate Reader, Agilent Technologies, Santa Clara, California). Results were normalized to a DMSO‐vehicle control, and growth curves were visualized using GraphPad Prism 10 (San Diego, CA, USA). Each cyanopeptide was tested in duplicate on each plate, and each experiment was repeated on different days to ensure technical and biological replication. All data are shown in Tables [Link], [Link]. Methods used for this process are visualized in Figure 2.

### 2.7 Cell Line Exposures to Combinations of Cyanopeptide Standards

Cells were seeded as above and allowed to adhere for 24 h. The activity of MC‐LA and AP‐B in combination was evaluated using a checkerboard assay [60]. Cyanopeptides were each four‐fold serially diluted in 50 μL of culture medium within four separate flat‐bottom microtiter plates starting at 100 μM. AP‐B was transferred to wells containing MC‐LA, so each row contained a serial dilution of MC‐LA and each column contained a serial dilution of AP‐B. Additionally, each plate included wells containing only single cyanopeptides, serially diluted four‐fold. The plates were incubated and read as above. The combination was tested in duplicate on each plate, and the experiment was repeated using different cell culture stocks to ensure technical and biological replication. All data are shown in Table S2.

### 2.8 Quantifying Cyanopeptide Combination Activity

SynergyFinder+ [61] was used to quantify the activity of MC‐LA and AP‐B in combination relative to their individual effects. The tool provides four scoring metrics to calculate the synergy score of an experiment based on specific assumptions: Bliss Independence (Bliss) [62], Loewe additivity (Loewe) [63], Zero Interaction Potency (ZIP) [64], and Highest Single Agent (HSA) [65] (Table S3). The Loewe additivity model was used because it defines the expected effect of a combination as if a molecule were combined with itself and considers the dose–response curves of individual molecules. The expected effect (*y*_loewe_) must satisfy Equation (1), where *x*_1_ and *x*_2_ are doses for each molecule and *X* ^1^_loewe_ and *X* ^2^_loewe_ are the doses of molecules 1 and 2 alone that produce *y*_loewe_.

(1) $$\left(\frac{x_{1}}{X_{\text{loewe}}^{1}}\right)+\left(\frac{x_{2}}{X_{\text{loewe}}^{2}}\right)=1$$

Using 4‐parameter log‐logistic (4PL) curves to describe dose–response curves, the parametric form of Equation (1) is derived in Equation (2), where *E*_min_ and *E*_max_ are the minimal and maximal effects of the molecules, *m*_1,2_ are the doses of the molecules required to produce the midpoint effect of *E*_min_ + *E*_max_(EC_50_), and *λ*_1,2_ are the shape parameters indicating the slope of the dose–response curves.

(2) $$\left(\frac{x_{1}}{m_{1}{\left(\frac{y_{\text{loewe}}-E_{\min}^{1}}{E_{\max}^{1}-y_{\text{loewe}}}\right)}^{1/\lambda_{1}}}\right)+\left(\frac{x_{2}}{m_{1}{\left(\frac{y_{\text{loewe}}-E_{\min}^{2}}{E_{\max}^{2}-y_{\text{loewe}}}\right)}^{1/\lambda_{2}}}\right)=1$$

A Loewe score greater than one is indicative of antagonistic effects, a Loewe score less than one is indicative of synergistic effects, and a Loewe score approximately equal to 1 is indicative of additive effects.

### 2.9 Cell Line Exposures to Culture Extracts

Extracts were reconstituted in 50/50 DMSO/water at a concentration of 100 mg/mL, based on the crude extract weight of the sample and stored at −20°C for the duration of experimental use. The viability and toxicity of all extracts were assessed in Hep‐3B, A549, and HK‐2 cells as above. Each extract was tested in duplicate on each plate, and each experiment was repeated on different days to ensure technical and biological replication. All data are shown in Table S4. Methods used for this process are visualized in Figure 2.

### 2.10 Statistical Analysis

Statistical analysis was performed using GraphPad Prism 10 (San Diego, CA, USA) and R (RStudio) using the *stats* package [66]. For comparisons involving less than two groups, a one‐way analysis of variance (ANOVA) was performed and followed up with Tukey's honest significant difference (HSD) multiple comparisons post hoc tests when applicable. All growth curve data are presented as the mean and standard error of the mean. A *p* < 0.05 was considered statistically significant in this study. To assess the relationship between cyanopeptide concentration in *Microcystis* culture extracts and cell viability, Pearson correlation coefficients (*R*) were calculated between cell viability at four doses (12.5, 25, 50, and 100 μM) and parts per billion (ppb) of each cyanopeptide congener. The amount of each cyanopeptide congener present in the 100 μM dose in the cell viability assays was calculated using Equation (1) in Table S5.

### 2.11 Figure Rendering

All figures were rendered in GraphPad Prism 10 software (San Diego, CA, USA) and R (RStudio) using the package *ggplot2* [66, 67].

## 3 Results

### 3.1 Dose‐Dependent Effects of Individual Cyanopeptides Across Different Cell Lines

**FIGURE 3:**
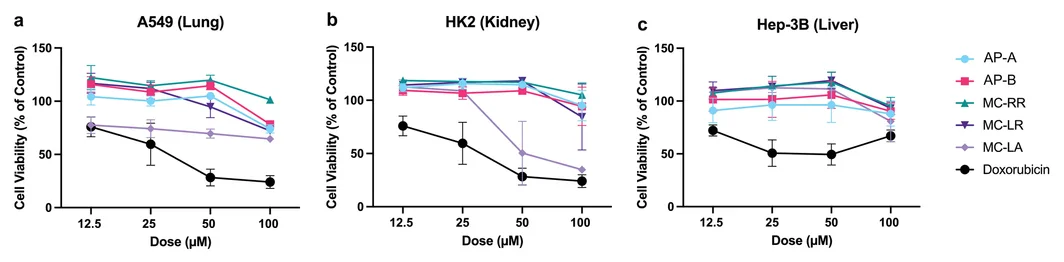
Dose‐dependent cell viability response by human (a) lung (A549), (b) kidney (HK2), and (c) liver (Hep‐3B) cells to pure MC and AP congeners. Viability is shown relative to DMSO (negative control) and compared to doxorubicin (positive control, data shown in black).

A549 (lung), HK2 (kidney), and Hep‐3B (liver) cells exposed to 0–100 μM of MC‐RR, MC‐LR, MC‐LA, AP‐A, and AP‐B for 48 h showed decreases in cell viability in a cell line‐, congener‐, and dose‐dependent manner (Figure 3) relative to a vehicle control (0.5% DMSO). The cyanopeptides did not significantly reduce cell viability across the concentrations as compared to the positive control (doxorubicin). A549 cell viability decreased most strongly in response to the individual cyanopeptides, followed by less pronounced effects in HK2 and Hep‐3B cells. Across all three cell lines, MC‐LA consistently caused the greatest decrease in viable cells. MC‐LR, AP‐A, and AP‐B exhibited similar levels of cell viability reduction, while MC‐RR induced the smallest decrease in cell viability out of all congeners tested across all cell lines. Significant decreases in cell viability by MC‐LA were observed at 100 μM in all cell lines, at 50 μM in both A549 and HK2 cells, and at 12.5 and 25 μM in A549 cells (*p* < 0.05, Tukey's HSD). Additionally, all cyanopeptides except MC‐RR significantly reduced viability at 100 μM in A549 cells (*p* < 0.05, Tukey's HSD).

### 3.2 Dose‐Dependent Effects of Cyanopeptide Combinations Across Different Cell Lines

**FIGURE 4:**
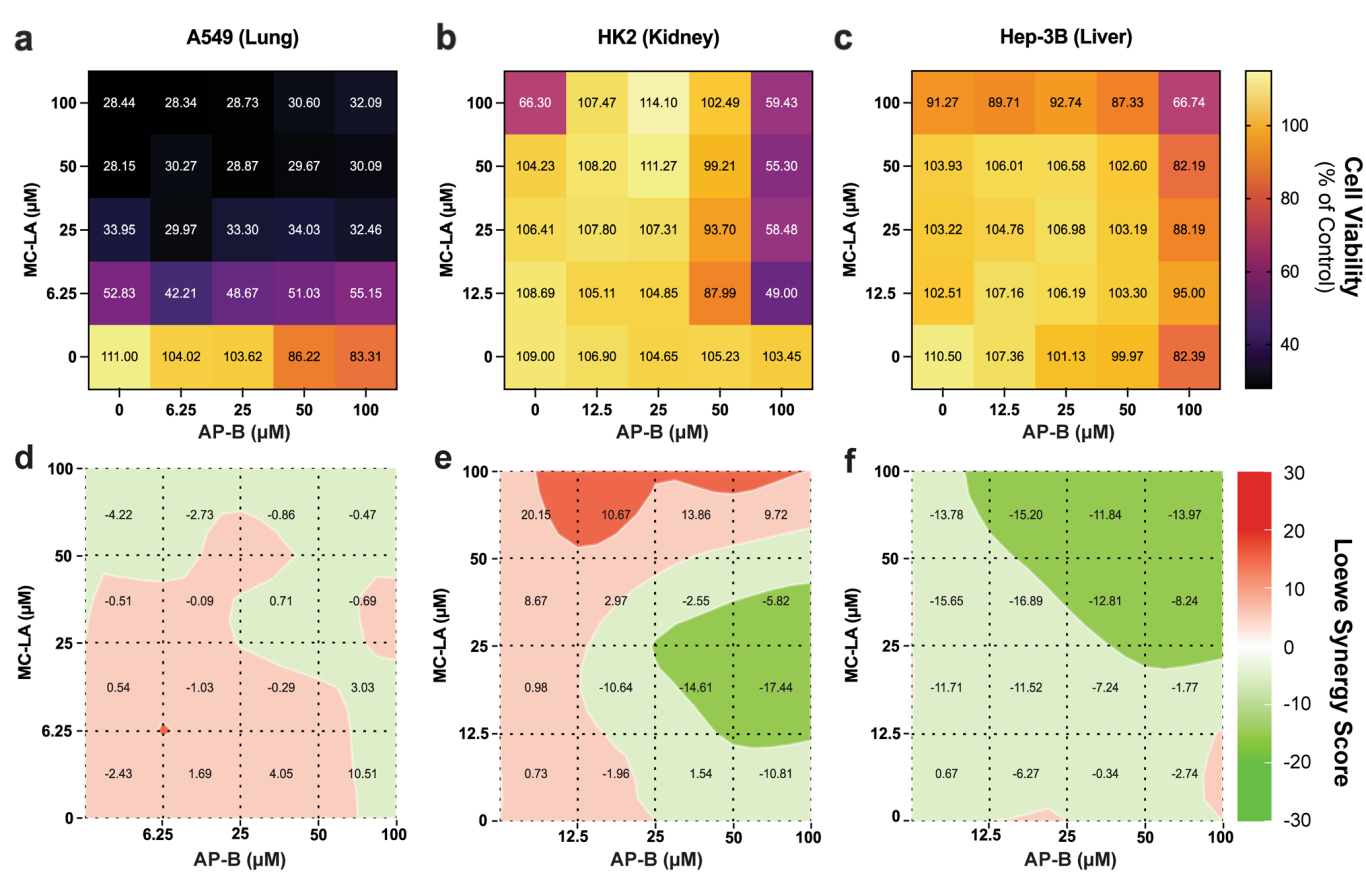
Dose‐dependent cell viability response by human (a) lung (A549), (b) kidney (HK2), and (c) liver (Hep‐3B) cells to combinations of MC‐LA and AP‐B. Viability is shown relative to DMSO (negative control), with lighter tiles indicating more cell viability and darker tiles indicating lower cell viability. 2D contour plots show Loewe synergy scores for MC‐LA and AP‐B combinations in (d) A549, (e) HK2, and (f) Hep‐3B cells. Scores < 1 indicate synergistic effects (green), ~1 indicate additive effects (white), and > 1 indicate antagonistic effects (red). Actual Loewe scores are displayed within each plot. Mean Loewe scores for each cell line are: A549: 0.45, HK2: −0.48, and Hep‐3B: −9.33.

The effects of MC‐LA, the most toxic MC congener, and AP‐B on cell viability were assessed in combination via checkerboard assays in A549, HK2, and Hep‐3B cells (Figure 4a–c). Loewe synergy scores for each combination were calculated and visualized (Figure 4d–f, Table S3), with scores < 1 indicating synergism (enhanced activity), > 1 indicating antagonism (decreased activity), and ≈1 indicating additivity. The combined exposure to MC‐LA and AP‐B resulted in dose‐dependent patterns of cell viability reduction that varied across all three cell lines.

In Hep‐3B cells, the synergistic effect of MC‐LA and AP‐B was the most consistent of all the cell lines. Cell viability was most greatly reduced under combined exposure to 100 μM MC‐LA and AP‐B, with increased cell viability observed with decreasing concentrations of both cyanopeptides. The mean Loewe synergy score for all combinations indicated synergism (−9.33), suggesting that combined exposure to both cyanopeptides increased cell viability reduction beyond their individual effects. Synergism was most pronounced at high combined doses (50–100 μM) and decreased in a dose‐dependent manner with concentration of both cyanopeptides.

In A549 cells, the greatest decrease in viable cells occurred following individual exposure to 100 μM MC‐LA. Similar effects were observed for combinations of high doses, between 50 and 100 μM, of MC‐LA and AP‐B. Mild effects on cell viability were observed at the lowest dose of MC‐LA. Dose‐dependent cell viability was observed for AP‐B, although maximum cell viability decreased with high doses of MC‐LA regardless of the presence of AP‐B. The mean Loewe synergy score for all combinations in A549 cells was 0.45, indicating neither strong synergism nor antagonism. Loewe synergy scores increased with higher concentrations of AP‐B in the presence of 100 μM MC‐LA, indicating less synergism and suggesting that AP‐B may interfere with the toxic effects of MC‐LA at high concentrations in this model.

In HK2 cells, cell viability decreased most following individual exposure to solely 100 μM MC‐LA and by MC‐LA between 12.5 and 100 μM when combined with 100 μM AP‐B. However, combinations of 100 μM MC‐LA and intermediate concentrations of AP‐B (12.5–50 μM) did not reduce cell viability. The mean Loewe synergy score for all combinations in HK2 cells was −0.48, not indicative of strong synergistic or antagonistic mechanisms. However, lower scores (synergistic) were observed at doses of 50 μM or lower of MC‐LA in combination with doses between 25 and 100 μM AP‐B, indicating strong synergism at these doses.

### 3.3 Dose‐Dependent Effects of Microcystis Culture Extracts Across Different Cell Lines

**FIGURE 5:**
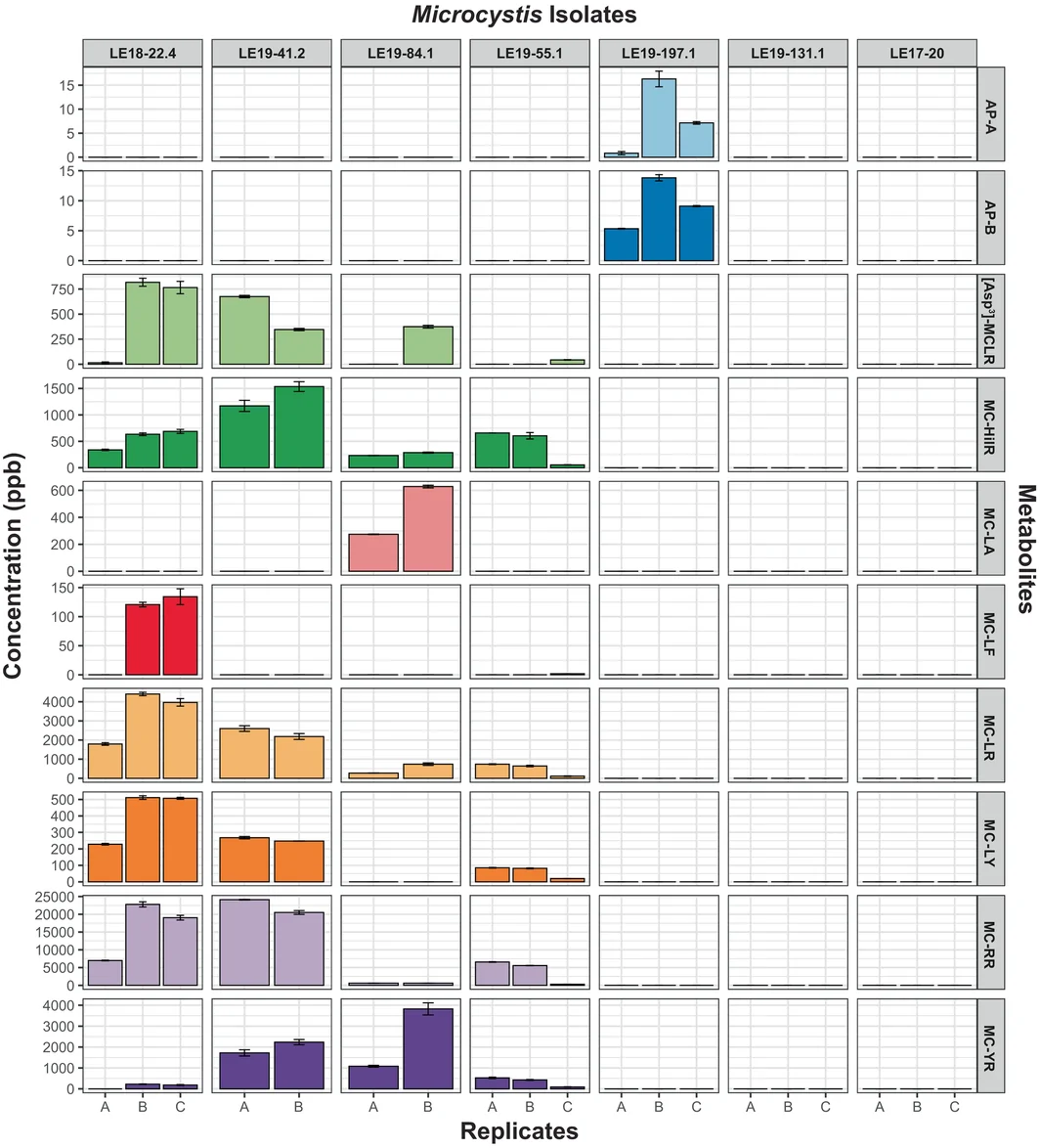
Concentrations of select congeners of microcystins and anabaenopeptins in crude extracts from WLECC *Microcystis* cultures used in human cell line exposures. Biological duplicates or triplicates are shown for each isolate. Bars represent the mean concentration in parts per billion (ppb) for each cyanopeptide, with standard error indicated with error bars.

**FIGURE 6:**
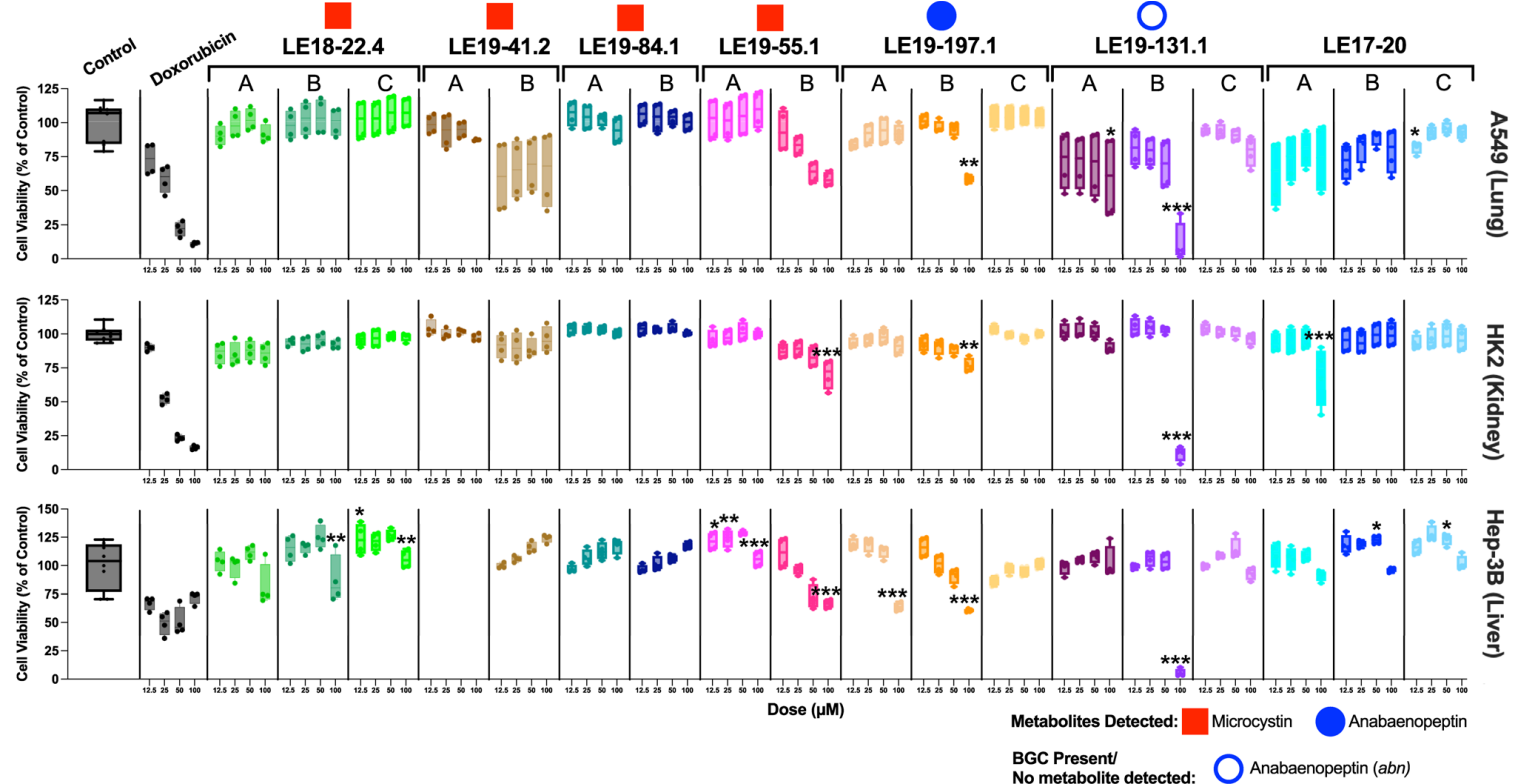
Dose‐dependent cell viability response by human lung (A549), kidney (HK2), and liver (Hep‐3B) cells to *Microcystis* isolate extracts from WLECC. Extracts were dosed between 12.5 and 100 μM, based on the crude dry weight of extracted metabolites. Each strain was grown in biological duplicate or triplicate, and cell viability was assessed in technical quadruples across two plates. Viability is shown relative to DMSO (negative vehicle control). A red square indicates the detection of microcystins in the extract, and a blue circle indicates the detection of anabaenopeptins in the extract. An unfilled blue circle indicates the presence of the anabaenopeptin biosynthetic gene cluster (BGCs) but no anabaenopeptin A or B detection. Significant effects were determined by Tukey's multiple comparisons post hoc tests against the vehicle control (*p < 0.01, **p < 0.001, ***p < 0.0001).

Extracts from seven *Microcystis* cultures were assessed for their cyanopeptide content (Figure 5) and dose‐dependent effects on cell viability across three cell lines (A549, HK2, Hep‐3B) (Figure 6). MCs and APs were never detected together in the same culture extract, and each strain produced only a subset of the measured congeners. The rarest MC congeners were MC‐LA, detected only in LE19‐84.1, and MC‐LF, present in LE19‐55.1 and LE18‐22.4. APs were only detected in extracts from LE19‐197.1, containing both AP‐A and AP‐B, while LE17‐20 and LE19‐131.1 had no detectable cyanopeptides. Some biological replicates from the same culture varied, including LE18‐22.4 (A vs. B and C), LE19‐55.1 (C vs. A and B), and LE19‐84.1 (A vs. B). This could be due to variable BGC expression and metabolite production across replicates of the same culture.

Culture extracts caused dose‐dependent reductions in cell viability across all three cell lines (Figure 6, Figure S1). At 100 μM of crude extract dissolved in a 50/50 mixture of DMSO and water, significant decreases in viability relative to vehicle controls were observed for LE19‐131.1B, LE19‐197.1B, and LE19‐55.1B in all three cell lines (*p* < 0.05, Tukey's HSD). Actual concentrations of MCs and APs present in these extracts at 100 μM doses ranged between 0.1 nM and 0.2 μM (Table S5), orders of magnitude lower than the concentrations used in the present study's individual cyanopeptide experiments (Figure 3). However, of these extracts, LE19‐131.1B had no detectable microcystins or anabaenopeptins within the extracts but caused greater reductions in viable cells than pure MC‐LA at 100 μM, suggesting the potential role of unidentified bioactive compounds or interactions within complex extract mixtures. Moreover, all LE17‐20 replicates, lacking any detectable MCs or APs, induced significant viability reductions across several doses in all cell lines (*p* < 0.05, Tukey's HSD). The most significant viability reductions were observed in Hep‐3B cells with 12 significant effects by different cultures, replicates, and doses (*p* < 0.05, Tukey's HSD), compared to four significant effects in both A549 and HK2 cells. These results indicate variability in extract toxicity based on culture, replicate, dose, and cell line (Figure S2).

**FIGURE 7:**
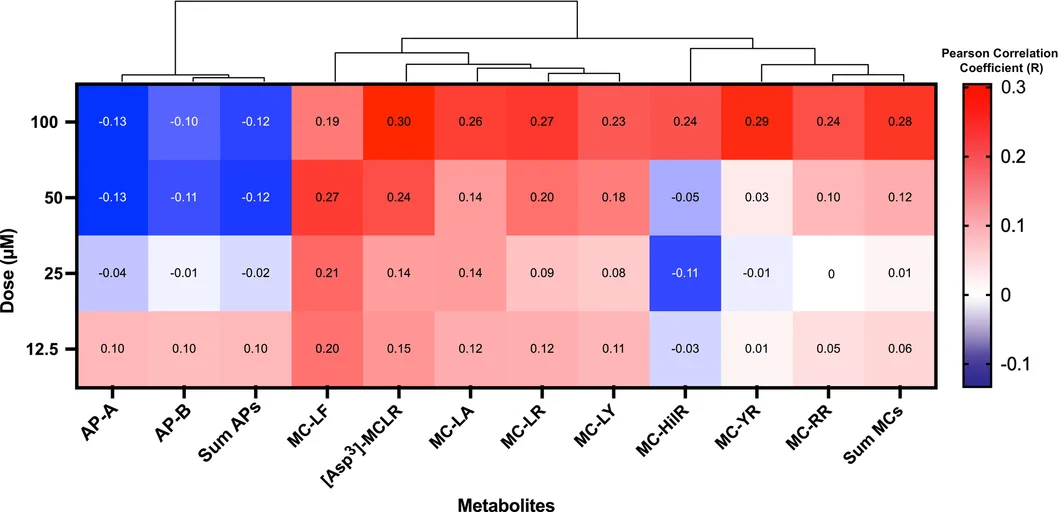
Pearson correlation coefficients (*R*) between cell viability effects exhibited by WLECC *Microcystis* isolates at four doses between 12.5 and 100 μM and cyanopeptide concentrations (MC and AP congeners) in each extract. Each tile shows the correlation coefficient (*R*), colored by strength and direction (blue (negative) to red (positive)). “Sum APs” indicate the sum of all AP congeners measured in the extract, and “Sum MCs” indicate the sum of all MC congeners measured in the extract.

To evaluate whether cyanopeptide content accounted for the variability in toxicity, Pearson correlation coefficients were calculated between cyanopeptide concentrations (ppb) and cell viability across four doses for the extracts (Figure 7). Weak correlations (*R* = −0.1 to 0.3) were observed between individual congeners, total MC or AP levels, and viability outcomes. MC content and viability correlations increased at higher doses (50–100 μM), while AP content and viability correlations decreased. Consistently low *R* values suggest that other factors, such as additional metabolites in the extracts and their interactions, may better explain observed viability outcomes.

## 4 Discussion

Across the globe, cyanobacteria are producing cyanotoxins, bioactive cyanopeptides, and a vast array of uncharacterized secondary metabolites in freshwater systems relied upon by millions of people, animals, and ecosystems [1, 4, 17]. These chemical cocktails pose an emerging threat in increasingly eutrophic waters. While regulatory frameworks have focused on a limited subset of well‐characterized cyanotoxins, the potential toxicity of unmonitored metabolites and synergistic toxicity from interactions among co‐occurring metabolites remains poorly understood. To address this gap, we evaluated the cytotoxic effects of two of the most commonly detected and abundant cyanopeptide classes in freshwater ecosystems, MCs and APs [8, 42], both individually, in combination, and within complex *Microcystis* metabolite mixtures using human lung, kidney, and liver cell lines.

**FIGURE 8:**
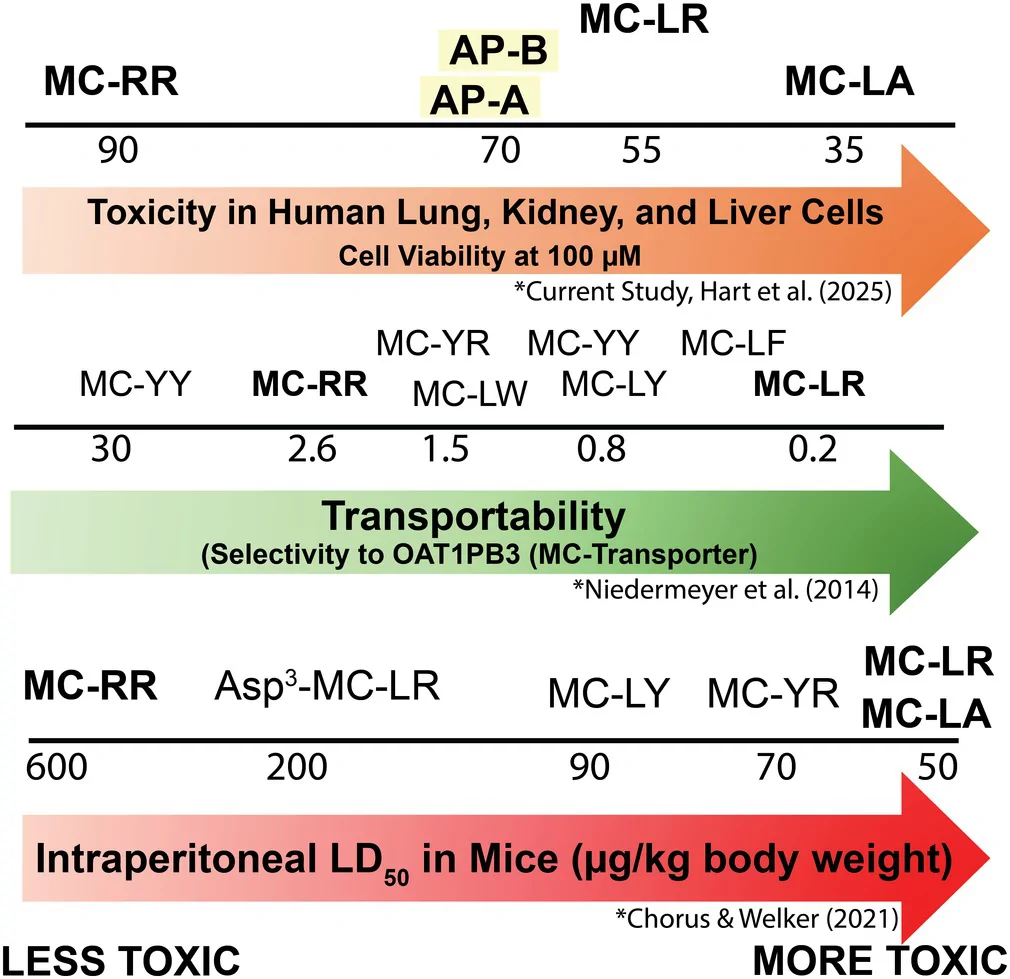
Comparative toxicity and transportability of microcystin and anabaenopeptin congeners. Percent cell viability at 100 μM for three microcystin and two anabaenopeptin congeners in human lung (A549), liver (HK2), and kidney (Hep‐3B) cells from the current study (orange arrow). OATP1B3 selectivity, a key transporter for MC‐LR, from Niedermeyer et al. [30] is indicated with a green arrow for eight microcystin congeners. Intraperitoneal LD₅₀ values (μg/kg) in murine models for six microcystin congeners are shown as summarized in Chorus and Welker [70] (red arrow). Congeners used in the current study are bolded. Congeners on the left tend to be less toxic across metrics, while those on the right tend to be more toxic. Protein phosphatase 1 and 2 inhibition across these congeners do not vary significantly [28, 71].

Consistent with previous studies on in vitro PP1/PP2A inhibition and in vivo toxicity [26, 68, 69, 70], MC‐LA was more toxic than MC‐LR, which was in turn more toxic than MC‐RR. Although all three congeners exhibit similar low‐nanomolar IC_50_ values against PP1/PP2A [68, 69], their effects on human cell viability vary substantially, from 35% to 90% reduction at 100 μM in the present study (Figure 8). This extensive variability in congener‐specific effects on human cell viability is consistent with murine intraperitoneal LD_50_ values, ranging from ~50 to 600 μg/kg^70^, and could reflect differences in transporter‐mediated uptake [29, 30, 71]. Studies using cells transfected with OATP1B1 and OATP1B3 show enhanced transporter selectivity for MC congeners containing leucine and acidic, aromatic residues in positions *X* and *Z* [30]. This aligns with observations that MC‐LW and MC‐LF exhibit highly potent IC50 values of ~0.4–0.6 nM in primary human hepatocytes, whereas MC‐RR is significantly less potent, with IC50 values ranging from 167 to 900 nM, representing a ~1000‐fold decrease in cytotoxicity [71]. MC congeners containing arginine in either the *X* or *Z* position (e.g., MC‐RR, MC‐LR) are less potent, possibly due to their dicationic nature at physiological pH, which may reduce OATP‐mediated uptake and thus, membrane transport [30]. These findings suggest that more toxic MC congeners such as MC‐LA, MC‐LW, and MC‐LF warrant increased monitoring and toxicological evaluation. Risk assessments that focus solely on MC‐LR may underestimate risk, particularly when more potent congeners are present. In western Lake Erie, a eutrophic basin of the Laurentian Great Lakes plagued by seasonal cyanoHABs, MC‐RR and MC‐LR are typically the dominant congeners, but MC‐LA concentrations rise later in the season and are the fourth most common MC congener throughout the basin. These results indicate the potential danger of microcystin congeners beyond MC‐RR and MC‐LR, warranting increased attention during the end of the bloom when attention and awareness of bloom conditions may decrease due to lower overall MC concentrations and less visible green scums [8, 72].

The MC congeners in this study exhibited variable toxicity across cell lines, with A549 and HK2 cells generally more affected than Hep‐3B cells. Unfortunately, these results cannot be directly compared to previous studies because to our knowledge, no studies have compared these specific congeners in A549, HK2, or Hep‐3B cells at high concentrations (> 50 μM). Differences between cell lines may stem from a combination of factors: variable expression of OATPs, differences in baseline metabolism, detoxification capacity, membrane composition and fluidity, susceptibility to stress, and intracellular accumulation [55, 56, 73, 74]. Among these, OATP expression is likely a major determinant. A549 and HK2 cells were more sensitive to MCs than Hep‐3B cells, which is consistent with reports that Hep‐3B cells express OATP1B1 and OATP1B3 at lower levels [55, 56, 73, 74]. Given that MCs are widely recognized for their hepatotoxicity, it is critical that the field adopts standardized hepatic cell models for evaluating congener‐specific toxicity mechanisms. For example, Hep‐G2 cells are commonly used in assessing the toxicity of MCs; however, they exhibit low OATP expression and may not accurately reflect MC uptake or toxicity [75, 76, 77]. To alleviate these issues, some studies recommend culturing hepatic cells in spheroid or aggregate formats to upregulate OATP expression and improve physiological relevance [78, 79]. Based on our findings, Hep‐3B cells may serve as a more responsive hepatic model for MC toxicity assessment, although their sensitivity could likely be enhanced through similar 3D culture methods.

Beyond hepatotoxicity, MCs also have nephrotoxic effects, including renal corpuscle enlargement, tubular damage, autophagy, and apoptosis [80, 81]. While Hek‐293 cells transfected with OATP1B1/1B3 are often used to study MC‐induced nephrotoxicity, we selected HK2 cells for their proximal tubule phenotype and endogenous OATP expression [30, 55, 71]. Our findings demonstrate that HK2 cells respond differentially to various MC congeners, supporting their utility as a physiologically relevant model for nephrotoxicity assessment of MCs. MCs have also been shown to exert cytotoxic and pro‐inflammatory effects in lung tissue, especially in A549 cells [38, 74, 82]. However, studies using A549 cells have primarily focused on the toxic effects of MC‐LR at lower concentrations (< 10 μM) [74]. The inclusion of additional MC congeners at high micromolar concentrations broadens the assessment of MC toxicity in pulmonary models and supports the use of A549 cells as a relevant in vitro system for evaluating MC‐induced effects in lung cell models. Based on this study and supporting literature, using OATP transporter‐expressing human cell lines is vital for understanding MC toxicity mechanisms [29, 30, 31, 71]. However, this focus introduces bias when studying other cyanopeptides like anabaenopeptins, whose uptake mechanisms remain unknown.

APs are the second most widely studied cyanopeptide class and are increasingly recognized for their high abundance in cyanoHABs, including those in the Laurentian Great Lakes and Mediterranean [8, 15, 42, 46]. APs often co‐occur with MCs and exhibit broad inhibitory effects of PP1/2A, serine proteases, and carboxypeptidases [15, 83]. In this study, AP cytotoxicity was greater than MC‐RR and comparable to MC‐LR. Despite this, APs are not currently regulated by the World Health Organization (WHO) or other agencies as MCs are. Given their widespread occurrence in water bodies used for drinking, recreation, and agriculture, our findings support the need to further study APs' effects on human cell lines, whole organs, and organismal models such as mice to establish health‐based guideline values for APs. Establishing such guidelines would not only enhance protection of humans and wildlife from cyanopeptide exposure but also drive further investigation into the mechanisms of action and cellular entry of APs, which remain poorly understood. Moreover, APs have been detected in water samples, aerosols, and fish tissues alongside MCs, highlighting the importance of considering their combined effects [41, 52, 84].

Given the frequent co‐occurrence of MCs and APs in cyanoHABs, we evaluated the combined effects of MC‐LA and AP‐B, the most potent congeners from each cyanopeptide class tested, across three human cell lines. In A549 cells, minimal synergy was observed, and no combination surpassed the cytotoxicity of MC‐LA alone at 100 μM. This may reflect the high expression of multiple OATPs in A549 cells, which facilitates efficient MC uptake and potent individual effects [73, 74]. MC‐LA and AP‐B may compete for similar transporters, as AP‐B shares a cyclic peptide scaffold, amphipathicity, and anionic charge at physiological pH with known OATP substrates [85]. However, its weaker inhibition of phosphatases may dampen combined effects [69, 86]. Alternatively, MC‐LA may dominate uptake and intracellular impact, leaving little opportunity for AP‐B to enhance toxicity unless dosed at higher concentrations. In HK2 cells, synergistic interactions were observed when MC‐LA was combined with high AP‐B concentrations. Unlike A549 and Hep‐3B cells, HK2 cells do not express OATP1B1 or OATP1B3, the main OATPs studied for MC transport, but they do express the kidney‐specific OATP4C1 [38, 55, 73]. Tissue‐specific transporter profiles likely shape these differential responses to MC and AP combinations [87].

Beyond differences in transporter profiles, kidney cells are highly sensitive to oxidative stress, which may be amplified by this co‐exposure [88]. Further research is needed to understand the effects of diverse cyanopeptides on toxicity, inflammation, and transporter regulation in kidney tissues. In Hep‐3B liver cells, strong synergy was observed with the combination of 100 μM MC‐LA and AP‐B. This may stem from liver‐specific enrichment of MC/AP targets like protein phosphatases and modest expression of OATP1B1/1B3, enabling co‐accumulation [89]. AP‐B may also potentiate MC‐LA toxicity by modulating stress pathways such as ROS or inflammatory signaling, although studies on AP bioactivity in liver cells are lacking. Taken together, these results raise the possibility that synergy between cyanopeptides depends not only on transporter availability but also on congener‐specific potency, intracellular signaling dynamics, and potential off‐target effects. Future studies should clarify the uptake mechanisms of APs, their targets in different tissues, and the broader cellular responses induced by co‐exposure.

In addition to MCs and APs, *Microcystis* can produce a chemically diverse repertoire of cyanopeptides and other secondary metabolites with known or predicted bioactivity [16, 17, 90]. Known bioactive cyanopeptides include serine protease and peptidase inhibitors such as aeruginosins, cyanopeptolins, microginins, and microviridins, and cyclamides [14]. Beyond these characterized molecules, *Microcystis* synthesizes many uncharacterized metabolites that may exert individual or combinatorial effects on human health [16, 91]. In this study, we assessed the cytotoxic effects of metabolite extracts from *Microcystis* cultures containing different profiles of known and unknown cyanopeptides and metabolites. The concentrations of MCs and APs in these extracts were poorly correlated with observed cytotoxicity at any given dose. For instance, LE18‐22.4 and LE19‐41.2 contained the highest concentrations of MCs but produced minimal effects on cell viability across all cell lines. In contrast, LE19‐55.1, which produces both MCs and the potent class of serine protease inhibitors, cyanopeptolins [16], showed clear cytotoxicity in all three cell lines. Similarly, LE19‐197.1 displayed strong cytotoxicity and was confirmed to contain APs.

The most toxic extract, LE19‐131.1, exhibited cytotoxicity exceeding that of MC‐LA alone at 100 μM, despite lacking detectable MCs or APs via targeted analysis. Genomic analysis indicates LE19‐131.1 harbors the biosynthetic potential to produce APs and cyanopeptolins, both of which can inhibit essential peptidases [16, 47, 92]. Because targeted analysis was focused on only two AP congeners, nontargeted metabolomics (Supporting Information: Note 1) was conducted and verified the absence of any AP congeners in all three LE19‐131.1 extracts. However, an ion with *m*/*z* 973.4, featuring fragments characteristic of cyanopeptolins including the 3‐amino‐6‐hydroxy‐2‐piperidone (Ahp) domain [93], was consistently detected across all biological replicates using nontargeted analysis, with the highest relative abundance in the most toxic replicate, B (Figure S3). While mass spectrometry alone cannot definitively identify this compound, the data support further investigation using a combination of bioassay‐guided and metabolomics‐guided discovery approaches to more precisely link chemical features with biological activity. Notably, cyanopeptolins are potent eukaryotic protease inhibitors, raising interest in this extract's toxicity despite the absence of APs and microcystins [94, 95].

Of the most toxic extracts, all except LE19‐55.1 encode the cryptic biosynthetic gene cluster *PmNT3*, predicted to produce a cyclophane‐like compound such as merocyclophane, a metabolite with known cytotoxic potential [16, 96]. Additionally, *Microcystis* produces non‐cyanopeptide bioactive metabolites such as pheophorbide, a chlorophyll breakdown product with pro‐apoptotic activity in the low micromolar range [97], which was previously detected in all cultures tested in this study [16]. *Microcystis* also synthesizes a diverse array of fatty acids and lipid derivatives [16, 98], which may contribute to cytotoxicity either directly or by enhancing the effects of co‐occurring toxins, potentially through membrane disruption or metabolite–metabolite interactions [99]. Consistent with other studies showing that *Microcystis* extracts lacking MCs can sometimes be more toxic than those with high MC concentrations [53, 100, 101, 102, 103], our findings support the hypothesis that other metabolites, either alone or in interaction, may be primary drivers of toxicity in some strains. Together, these results emphasize the need for comprehensive metabolomic and toxicological profiling of *Microcystis* strains beyond MC‐focused risk assessments. Uncharacterized or combinatorially acting metabolites may represent significant but overlooked contributors to the health risks posed by cyanoHABs.

## 5 Conclusion

This study demonstrates the cytotoxic effects of MCs and APs individually, in combination, and within complex *Microcystis* metabolite mixtures on human lung, kidney, and liver cell models. Although APs exhibited cytotoxicity comparable to some MC congeners and showed synergistic interactions with MCs across all cell lines, MC and AP concentrations within complex *Microcystis* mixtures were poorly correlated with overall cytotoxicity, suggesting the involvement of additional metabolites and interactive effects. These findings highlight APs as toxicologically relevant cyanopeptides that warrant further investigation and evaluation for regulation and routine monitoring, particularly given their high abundance in freshwater systems impacted by cyanoHABs. More broadly, this work contributes to a growing body of evidence that risk assessments focused solely on MCs may underestimate the full scope of toxicity in cyanoHABs [8, 14, 15, 42]. Reassessing our current toxicity frameworks to include a broader range of cyanopeptides and metabolite interactions is essential for accurately evaluating health risks and protecting both human and environmental health in the face of increasingly frequent and chemically diverse blooms.

## Funding

This work was supported by the National Institute of Environmental Health Sciences (5P01ES028939‐02), the National Science Foundation (OCE‐1840715), the National Institute of Health (1F31AI186432‐01 and 1F31ES036421‐01), and the Ohio Department of Higher Education.

## Conflicts of Interest

The authors declare no conflicts of interest.

## Supporting information


**Data S1:** tox70028‐sup‐0001‐Supinfo.docx.

**Table S1:** tox70028‐sup‐0002‐DataS1.xlsx.
**Table S2:** tox70028‐sup‐0002‐DataS1.xlsx.
**Table S3:** tox70028‐sup‐0002‐DataS1.xlsx.
**Table S4:** tox70028‐sup‐0002‐DataS1.xlsx.
**Table S5:** tox70028‐sup‐0002‐DataS1.xlsx.

## Data Availability

The data that support the findings of this study are available in the Supporting Information of this article.
