# Supplementary material for: Cyanopeptide Mixtures Induce Variable Synergistic and Antagonistic Effects Across Diverse Human Cell Lines
Source: Environ Toxicol. 2026 Jan 10;41(10):807–21. doi: 10.1002/tox.70028 (PMC13537360; doi:10.1002/tox.70028)
Supplement: Supplementary file 1 [file TOX-41-807-s001.docx]

**Supplementary Information**

**SI Table 1:** Single cyanotoxin exposure viability data.

**SI Table 2:** Combination cyanotoxin exposure viability data.

**SI Table 3:** Combination cyanotoxin exposure Loewe, Bliss, HAS, and ZIP synergy scores.

**SI Table 4:** *Microcystis* isolate extract exposure viability data.

**SI Table 5:** Concentration of cyanotoxins detected via targeted LC-MS in each crude extract in the 100 µM dose exposed to each cell line in viability experiments.

**SI Figure 1:** Mean viability and ±95% confidence interval of (a) lung (A549), (b) kidney (HK2), and (c) liver (Hep-3B) cell lines following exposure to four doses (100, 50, 25, 12.5 µM) crude extracts of *Microcystis* isolates from the WLECC relative to the DMSO vehicle control (shown in black). The dotted line represents the mean viability of DMSO vehicle controls. Isolate effects below the dotted line indicate significant cytotoxicity relative to control.

**SI Figure 2:** Principal Coordinates Analysis (PCoA) of viability responses to WLECC *Microcystis* isolate extracts in human cell lines lung (A549), liver (Hep-3B), and kidney (HK2), based on Euclidean distances of mean viability. Each point represents one isolate–cell line combination, colored by isolate identity. Shapes and sizes of points vary across panels where in **(a)** shapes indicate the cell line tested, **(b)** point size is scaled to total microcystin content (ppb) in the extract, and **(c)** point size is scaled to total anabaenopeptin content (ppb) in the extract. PCoA axes represent the proportion of total variance explained (PCoA 1: 66.8%, PCoA 2: 28.3%).

**SI Figure 3: (A)** Total ion chromatograms (TICs) of samples 3A, 3B, and 3C from LE19-131.1. were obtained using Q-ToF mass spectrometry, each analyzed in technical triplicate (as described in SI Note 1). A consistent peak at 5.5 minutes, highlighted by the black box, was detected across all three culture samples and their corresponding technical replicates. **(B)** Electrospray Ionization Mass Spectrometry (ESI-MS) analysis at 5.5 minutes for sample 3B reveals a dominant ion at *m/z* 1031.4268. An associated ion, [M+H_2_O+H]^+^, at *m/z* 991.4332 is identified as an artifact of the dominant peak with the ion at *m/z* 973.4236. The ion 973.4236 *m/z* consistently appears across all biological samples (C3A–C3C) and their technical replicates and yields a well-defined MS² fragmentation pattern, making it a candidate for structural characterization and downstream analysis. **(C)** Extracted Ion Chromatogram (EIC) for samples 3A, 3B, 3C, and the methanol extraction control reveals the 973.4246 *m/z* ion in the culture samples (3A-C) and no abundance of the ion in the methanol blank control. **(D)** MS^2^ fragmentation of the 973.4 *m/z* ion indicates the presence of fragments 215.1192 *m/z*, 243.1137 *m/z*, and 454.1534 *m/z* which correspond to the fragments [Ahp-Phe-H_2_O-CO+H]^+^, [Ahp-Phe-H_2_O+H]^+^, and [Ahp-Phe-Cl-N*Me*Tyr -H_2_O+H]^+^, respectively. These diagnostic ions support the presence of the Ahp (3-amino-5-hydroxy-6-piperidone) moiety and are consistent with the micropeptin/cyanopeptolin class of cyclic peptides, potentially representing Cyanopeptolin 990B. However, further validation is required to identify the compound. See SI Note 1 for citations regarding this annotation.

**SI Note 1: Non-targeted metabolomics analysis**

The samples used in this analysis were prepared from crude extract weight and reconstituted in methanol spiked with 10 µg/uL of liquid chromatography-mass spectrometry (LC-MS) caffeine standard kit (Part Number 8500-6917). Extracts were centrifuged at 17,000 rpm for 20 minutes and supernatants were collected for further dilution of samples using methanol/caffeine stock to a concentration of 0.2 mg/ml after 10-minute centrifugation at 17,000 rpm. Samples were analyzed on the high-performance liquid chromatograph quadrupole time-of-flight (Q-ToF) mass spectrometer equipped with ESI source in positive mode. Total injection on the Q-ToF machine was 2 µL of sample at a concentration of 0.2 mg/mL. Chromatographic separation was performed using Agilent Infinity Lab Poroshell 120 EC-C18 column 2.1 x 50 mm, 1.9 µm column at a flow rate of 0.35 mL/min.

The gradient mixture of solvent A (100% H_2_O with 0.1% formic acid) and B (95% acetonitrile, 5% H_2_O with 0.1% formic acid) was the following: 0-1minute 10% B, 1–4 minute linear gradient to 35% B, 4-8 minute linear gradient to 80% B, 8–10 minute linear gradient to 100% B with maximum pressure limit 1000 bar. Gas temperature was set to 325C° with drying gas parameters set to 11 l/minute. Nebulization gas pressure was set to 35 psi. Sheath gas temperature parameters were set to 350C° with sheath gas flow of 11 L/minute.

Data were collected from *m/z* 100 to 2000 with acquisition rate of 2 spectra/second, an absolute threshold of 500, and relative threshold of 0.01% in positive ion mode. Auto MS/MS scan fragmentation was collected with a mass range from 50-2000 m/z with the acquisition rate of 6 spectra/second and 166.7 ms/spectrum. The MS/MS data were analyzed using a maximum of 9 precursors per cycle with the precursor threshold parameters set to 200 count for absolute threshold and 0.01% relative threshold. Iterative MS/MS was included allowing for a mass error tolerance of 20 ±1 ppm and retention time exclusion tolerance of 0.2 minutes. A blank sample was run initially to curate an exclusion list. Excluded ions were 437.1939, 284.3323, 274.2744, 301.2849, and 385.3795 *m/z*.

All data was visualized and analyzed in Agilent MassHunter Qualitative Analysis v10.0. After identifying peak differences in MS and MS/MS data, we identified fragmentation patterns appearing consistently in the technical replicates that differed from the extraction control. Fragmentation patterns present in the MS/MS data matched with reported fragmentation patterns of cyanopeptolins/micropeptins (1). Conversely, we were unable to identify diagnostic fragmentation patterns consistent with anabaenopeptins (2). Extracted ion chromatograms of diagnostic m/z confirmed the presence of the ions in the 3A-C3 cultures run in triplicate and no abundance of these diagnostic ions were detected in the methanol extraction control.

1.	McDonald K, Renaud JB, Pick FR, Miller JD, Sumarah MW, McMullin DR. Diagnostic fragmentation filtering for cyanopeptolin detection. Environmental Toxicology and Chemistry. 2020 Nov 25;40(4):1087–97.

2.	Welker M, Maršálek B, Šejnohová L, von Döhren H. Detection and identification of oligopeptides in *Microcystis* (cyanobacteria) colonies: Toward an understanding of metabolic diversity. Peptides. 2006 Sep 1;27(9):2090–103.
